# Enhancing faba bean (*Vicia faba*) productivity under drought stress through modulation of physiological traits and antioxidant enzyme system using thiourea and hydrogel

**DOI:** 10.1186/s12870-025-06904-0

**Published:** 2025-07-04

**Authors:** Amany A. Ramadan, Mohamed F. El-karamany, Maha M. S. Abdallah, Bakry A. Bakry, Hala M. S. El-Bassiouny

**Affiliations:** 1 Botany Department, Agricultural and Biological Research Institute, National Research Centre, 33 El Bohouth Street, Dokki, P.O. 12622, Giza, Egypt; 2 Field Crops Research Department, Agricultural and Biological Research Institute, National Research Centre, 33 El Bohouth Street, Dokki, P.O. 12622, Giza, Egypt

**Keywords:** Faba bean, Drought stress, Thiourea, Hydrogel, Osmoprotectants, Antioxidant enzymes

## Abstract

**Background:**

Due to limited water supplies and rapid population growth, most countries are facing significant challenges with agricultural output. Most efforts have concentrated on utilizing soil additives to enhance soil properties and boost water use efficiency. The purpose of this study was to assess the impact of thiourea and hydrogel applications on faba bean (*Vicia faba*) plants under water shortage conditions. Two field experiments were conducted to study the effects of thiourea at concentrations of (200, 400, and 600 mg/L) and hydrogel soil amendments at rates of (0 and 75 kg/ha) on faba bean plants under 100% (5950 m^3^/ha/season) and 50% (2975 m^3^/ha/season) of irrigation water requirements (IWR) in sandy soil.

**Results:**

The findings revealed that an irrigation water shortage (50%) of irrigation water requirements; induced significant reductions in all growth parameters, photosynthetic pigments, and yield in contrast with 100% IWR. The seed yield was lowered by 35.91 and 32.54% at 50% IWR without and with hydrogel addition, respectively. However, the external application of thiourea significantly mitigated the negative effects of water shortage. The plants treated with thiourea showed increases in all growth criteria, photosynthetic pigments, compatible solutes, IAA, and phenolics and also improved the activities of peroxidase (POX), polyphenol oxidase (PPO) and superoxide dismutase (SOD) as compared with untreated plants. Also, the amendment of hydrogel to soil mitigates all tested parameters. The amount of absorbed water increased by 39.68 and 45.53% with thiourea (400 mg/L) in the absence and presence of hydrogel, respectively as compared with the control (50% IWR). In general, the application of 400 mg/L thiourea with hydrogel (75 kg/ha) induced the most pronounced increase in seed yield (quantity and quality) and its attributes.

**Conclusion:**

Under drought conditions (50% IWR), foliar spraying with thiourea and hydrogel amendment to sandy soil not only positively affected water potential, osmolytes, chlorophyll and carotenoids contents, but also significantly changed the activities of antioxidant enzymes that enabled faba bean plants to withstand drought and increased productivity.

## Introduction

Water scarcity is known to induce drought stress which is considered one of the major problems for crop production in the world and in Egypt. This needs to either reducing irrigation water consumption via conservation of the mass of water around crop root zone or keeping the soil-water balance, especially in sandy soil conditions. Therefore, scientists have directed their attention towards finding solutions to overcome poor soil characteristics especially in these new expansion areas. Irrigation water deficit is one of the limiting criteria for plant growth and photosynthetic pigments responsible for lowering the production of commercial crops including rice, wheat, maize, and faba beans worldwide [1]. Drought (an abiotic stress factor) is associated with global warming and is considered a major environmental stress that affects plant growth [2]. Drought stress indicators refer to oxidative damage related to the injuries caused by various biomolecules like proteins, which affects the plant’s metabolism and limits its growth and productivity [3] characteristics, especially in these new areas.

Thiourea is a sulfur-rich plant growth promoter with 42% sulfur (S) and 36% nitrogen (N) that efficiently inhibits oxidative stress produced by abiotic stressors on plants as well as modulating plant development, since it is considered a non-physiological thiol-based ROS scavenger [4]. It can reduce redox imbalances induced by stress and other plant injuries [5]. Its principal functional groups, amino (NH_2_) and thiol (SH), are thought to be crucial for the molecule’s ability to respond to oxidative stress and, especially, to fulfill the increased need for nitrogen in the event of abiotic stress [6]. It has been proven to be efficient in reducing crop stress induced by drought [7]. Among other biochemical alterations in many plant species, thiourea seed treatment increased antioxidant production and ultimately resulted in decreased production of malondialdehyde and hydrogen peroxide in salt-grown *B. juncea* [8] and mung bean [9]. Thiourea application increased antioxidant enzyme activity and improved the levels of phosphorus, potassium, calcium, magnesium, and carotenoids in the leaves of wheat plants [10].

Hydrogel keep water accessible and stagnant beside plant roots, and hence minimizing its loss due to evaporation and penetration in the soil to provide large quantities of water irrigation. As evident from the literatures, hydrogel is a super absorbent polymer (SAP) that can be considered as one of the promising strategies to reduce drought stress [11]. Particularly in dry and semi-arid regions, hydrogels has been found to be an efficient method for improving the soil’s ability to retain water and preserve moisture [12]. These cross-linked compounds make up hydrophilic gels (Polymers), which absorb water without decaying. Guilherme et al. [13] stated that it increases the absorption capacity (g water g^−1^ soil) of water up to 400–2000 times its dry weight which reduces water stress for plants. Tariq et al. [14] pointed out the benefits of hydrogels to the soil in improving soil porosity, water-holding capacity and aeration, the rate of infiltration, nutrient uptake, and water absorption. These tangible changes have been found to be reflected positively in promoting plant development and yield characteristics. Researchers from the National Research Center (NRC) in Egypt have shown that hydrogel can effectively reduce water irrigation for a variety of crops cultivated in Egypt by up to 50 or 75% of the optimum level in sandy soil [15]. Waly et al. [16] worked on potatoes and showed that hydrogel had a beneficial influence on the soil’s ability to retain water and use it more efficiently. Moreover, the use of hydrogel lowered the quantity of water utilized for irrigation and lowered the amount of nitrogen that leached from sandy soil [17].

Nowadays, faba bean (*Vicia faba* L.) is a major crop in Egypt, since it is a significant export good source of protein for both human and animal consumption [18]. The expansion of faba bean in newly reclaimed expansion soils faces the poor characteristics of these soils as well as the abiotic stress factors caused by irrigation water shortage in these areas. So, the search for solutions, e.g. soil fertility aids and/or irrigation water preservation tools, to overcome such threats became a must outside the traditional frame of solutions.

The present work was undertaken to improve plant defense mechanisms (osmoprotectants, indole acetic acide, phenols, antioxidant enzymes, and nutrient elements) through foliar sprays of thiourea at different concentrations and hydrogel addition to mitigate abiotic stress (specifically drought stress) and consequently improve growth, photosynthetic pigments, yield, and yield attributes of the faba bean plant.

## Materials and methods

### Experimental site

**Fig. 1:**
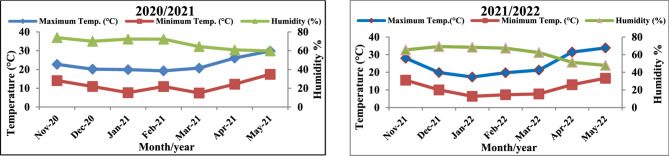
Means of temperature and relative humidity at the experimental area during the two studied seasons

On line with the increased population in Egypt with the limited area of cultivation, it became a must for the expansion in the desert areas to increase crop production. The challenge facing agricultural activities in the new areas is the nature of soil that cannot keep water requirements for plant growth due to high porosity particularly in sandy soils. So, this field experiment was carried out on November ^1^st in the 2020–2021 and 2021–2022 at the Research and Production Station of the National Research Centre (NRC), Al-Nubaria District, Al Behaira Governorate, Egypt (30^0^_86’67” N 31^0^_16’67” E) with a mean altitude of 21 m above sea level). The farm’s location is assumed to be either arid or semi-arid. The climate data for the experimental site during the growing season are displayed in Fig. 1.

The experimental soil was examined by Chapman & Pratt [19] before the addition of hydrogel treatments. Table 1 displays the properties of the sandy soil texture.

**Table 1:** Some physical characteristics, water relations, chemical and nutritional properties of the experimental soil at the end of 2020–2021 and 2021–2022 seasons

**Season**	**Some ** **physical characteristics of soil**				**Water relations**		
	**Constant Depth (cm)**	**Sand %**	**Silt %**	**Clay%**	**Field capacity % (by vol.)**	**Wilting Point % (by vol.)**	**Available water** **%**
**20** **20** **/202** **1**	00– 30	85.3	10.5	4.2	18.0	7.4	10.2
	30– 60	81.2	13.6	5.2	23.0	9.3	12.8
**202** **1** **/202** **2**	00– 30	81.3	13.4	5.3	42.6	38.7	13.7
	30– 60	74.6	17.6	7.8	38.1	36.5	17.8

	**Soil chemical and nutritional properties of the experimental site**													
	**Constant Depth (cm)**	**pH**	**Electrical conductivity (dS/m)**	**Saturation** **Percentage**	**Anions** **(Mill equivalents/liter)**				**Cations** **(Mill equivalents/liter)**			CaCo_3_ %		**Organic Mater %**
					CO_3_^--^	CO_3_-	**Cl**	SO_4_^--^	Ca^++^	Mg^++^	Na^+^	K^+^		
**20** **20** **/202** **1**	00– 30	7.84	1.17	32	--	0.50	8.40	1.11	1.80	0.90	7.1	0.20	1.00	0.40
	30– 60	7.89	1.79	27	--	0.60	8.00	1.40	2.10	1.50	6.2	0.20	6.00	0.07
**202** **1** **/202** **2**	00– 30	7.95	1.59	23	--	0.32	12.70	1.98	4.00	1.80	9.0	0.20	1.90	0.38
	30– 60	7.85	1.81	25	--	0.45	15.40	2.15	5.60	2.00	10.2	0.20	1.30	0.32

### Irrigation water requirements (IWR) for faba bean plants

According to Allen et al. [20], the Penman Monteith equation and crop coefficient were used to determine the daily actual irrigation water needs. All of the transpiration and evaporation differences between the two surfaces were merged into a single coefficient (Kc). The crop water requirements in the current study were calculated using the crop coefficient technique [21]. The amounts of regular irrigation water employed were 5950 (100%) and 2975 (50%) m^3^/ha/season IWR in both studied seasons. The typical quantity of water used for irrigation when using a drip irrigation system for both winter seasons 2020/2021 and 2021/2022, under each irrigation level, was calculated using the following equation:

$$\:IWR=\left(EToxKcx4.2\right)x1.2$$

Where: ETo: Reference evapotranspiration, Kc: Crop coefficient, 4.2 and 1.2 according to Allen et al. [20].

### Experimental design

The seeds used in these experiments were faba bean variety (Nubaria-1) obtained from the Agricultural Research Center in Egypt. The applied thiourea was obtained from Sigma-Aldrich Company, while the hydrogel used (a copolymer of sodium acrylamide-acrylate) is a commercially available soil improving product (Barbary) that is manufactured by a French company and registered with the French Ministry of Agriculture under number 9,010,133. It is also registered by the Egyptian Ministry of Agricultural Research Centre. It contains macro- and micronutrients (40% hydrogel polymer, 6.50% N, 4.80% P, 8.20% K) and has a holding- capacity of 300–500%. The hydrogel was added to the soil by spreading during land preparation. Its addition rate was 75 kg/ha before planting.

The design of the experiment was a randomized complete block design, arranged under a split-split plot design layout with three replicates. The irrigation water requirements (100 and 50%) occupied the main plots, whereas hydrogel at a rate of (0 and 75 kg/ha) were billed in sub-plots and the concentrations of thiourea at rates of 0, 200, 400 and 600 mg/L were allocated at random in sub-plots. The experimental plot measured 10.5 m^2^ (3 m long and 3.5-meters wide), with rows spaced 60 cm apart. Prior to planting, the ditches were treated with hydrogel at a rate of 0 and 75 kg/ha. The ditches were then filled in with soil to form rows. On both sides of the ridge, on November 1 st of each growing season, two seeds per hill (20 cm hill’s apart) were planted. During the seedbed preparation process, calcium superphosphate (15.50% P_2_O_5_) and potassium sulphate (48% K_2_O) were added at additional rates of 73.78 and 57.12 kg/ha, respectively. Ammonium nitrate (33.50% N), was added at a rate of 178.50 kg N/ha. Plants were watered every five days for two hours using a drip irrigation system that delivered water at rates of 100% and 50%.

After seeding, the plants were sprayed twice with freshly prepared thiourea solutions at concentrations of 200, 400, and 600 mg/L. This was done at 45 and 60 days after planting. In the meantime, as a control, distilled water was sprayed on the untreated plants. To measure growth parameters (plant height (cm), shoot fresh weight (g), shoot dry weight (g)) and the amount of water in the plant, the samples were collected at 75 days of age. At harvest, 10 plants from each plot were randomly selected, and the following parameters were noted: shoot length, plant weight (g), number of pods/plant, pods weight/plant, seeds number/plant, seed yield/plant (g), seed yield (Kg/ha), biological yield (Kg/ha) and straw yield (Kg/ha)]. Also, Water productivity (WP) is measured according to the following equation.

$$\text{WP = seed yield (kg/ha)/ irrigation water requirements} (\text{m}^{3}/ \text{ha}).$$

### Biochemical analysis

We assessed photosynthetic pigments (Chlorophyll a, chlorophyll b and carotenoids) using the Lichtenthaler and Buschmann [22] technique. Total Soluble Sugars (TSS) was extracted using the method by Gomez et al. [23] and identified by Albalasmeh et al. [24]. The Gusmiaty et al. [25] technique was used to extract and evaluate the indole acetic acid contents. The method for measuring phenolic content was as stated by Maurya and Singh [26]. The total soluble protein (TSP) was determined using Bonjoch and Tamayo’s [27] techniques. Proline content was extracted and determined by Tamayo and Bonjoch’s [28]. According to Kalsoom et al. [29], free amino acids (FAA) have been extracted and determined using the ninhydrin reagent technique by Verslues [30]. The macro-nutrient elements in faba bean seeds (N, P and K) were determined using the method of Cottenie et al. [31]. Carbohydrate % and the total values of total N were determined by AOAC [32].

According to the kind of enzyme, several assays were used to measure its activity. Enzyme extracts were made using Chen and Wang [33] methodology. The nitro-blue-tetrazolium reduction technique was used to determine the activity of superoxide dismutase (SOD, EC 1.12.1.1) [33]. The activity of peroxidase (POX, EC 1.11.1.7) was assessed by Kumar and Khan [34]. The activity of polyphenol oxidase (PPO) (EC 1.10.3.1) was determined by Cho and Ahn [35].

### Statistical analysis

The data were statistically analyzed on randomized complete block design (RCBD) arrangement in split-split plot layout with three replicates according to Snedecor and Cochran [36]. A combined analysis of the two growing seasons was carried out after testing the homogeneity according to Bartlett’s test which showed no significant differences between the two study seasons. Means were compared by using Duncan multiple range tests at 5% levels of probability [37]. Minitab [38] ver. 17.1.0.0 for Windows was used to analyze the results. Data were also subjected to principal component analysis (PCA) and Pearson correlation coefficient.

## Results

### Growth criteria

Table 2 presented the effects of the tested various thiourea concentrations on the growth of faba bean plants grown in the two irrigation water requirements (50 and 100% IWR) in the presence and absence of hydrogel.

**Table 2:** Impact of thiourea and hydrogel with water regime on morphological criteria of bean plants

IWR (%)	Thiourea (mg/L)	Plant height		Shoot FW		Shoot DW		Amount of water	
		Hydrogel -	Hydrogel +	Hydrogel -	Hydrogel +	Hydrogel -	Hydrogel +	Hydrogel -	Hydrogel +
**100**	**Control**	31.0 ± 0.58^h^	49.3 ± 1.20^de^	41.4 ± 0.21^f^	47.0 ± 0.30^e^	2.6 ± 0.13^i^	4.2 ± 0.08^d^	38.8 ± 0.12^e^	42.8 ± 0.20^de^
	**200**	48.3 ± 0.67^e^	52.6 ± 0.33^bc^	69.9 ± 0.44^c^	72.5 ± 0.45^b^	2.8 h ± 0.16^i^	6.5 ± 0.21^a^	67.1 ± 0.09^b^	66 ± 0.03^b^
	**400**	52.5 ± 0.67^cd^	66.0 ± 0.58^a^	71.7 ± 0.81^b^	74.8 ± 0.99^a^	4.7 ± 0.08^c^	6.00.09^b^	67 ± 0.17^b^	68.8 ± 0.2^a^
	**600**	54.6 ± 0.88b^c^	55.6 ± 0.88^b^	61.9 ± 0.93^d^	68.7 ± 0.64^c^	3.4 ± 0.21^fg^	5.8 ± 0.09^b^	58.5 ± 0.21^d^	62.9 ± 0.22^c^
**50**	**Control**	26.0 ± 1.73^i^	40.3 ± 0.33^fg^	27.4 ± 0.68^k^	33.6 ± 1.13^i^	1.7 ± 0.05^j^	3.2 ± 0.14^g^	25.7 ± 0.09^l^	30.4 ± 0.04^j^
	**200**	40.0 ± 2.31^fg^	43.8 ± 0.88^f^	38.0 ± 0.22^h^	40.0 ± 0.26^fg^	2.6 ± 0.06^i^	3.7 ± 0.09^ef^	35.4 ± 0.11^h^	36.3 ± 0.08^g^
	**400**	32.0 ± 1.00^h^	47.0 ± 1.15^e^	38.6 ± 0.63^gh^	41.3 ± 0.50^f^	2.7 ± 0.13^i^	3.9 ± 0.04^de^	35.9 ± 0.15^hi^	37.4 ± 0.21^fg^
	**600**	27.6 ± 0.33^i^	42.3 ± 1.20^fg^	30.9 ± 0.04^j^	38.4 ± 0.50^gh^	2.8 ± 0.11^hi^	3.10 ± 0.09^gh^	28.1 ± 0.13^k^	35.3 ± 0.01^i^

Data are means ± standard error (*n* = 3)

a, b, c,… Means with different letters in the same column are significantly differed at (*P* < 0.05)

(–) means absent of hydrogel, **(+)** means present of hydrogel

Drought stress (50%) in the soil induced a significant (*P* < 0.05) reduction in the tested morphological criteria (Plant height, shoot fresh and dry weights, and amount of water in shoot) in contrast to plants grown at the levels of 100% IWR. All foliar spraying of thiourea significantly (*P* < 0.05) increased the growth parameters of faba bean plants. Meanwhile, the plants cultivated in the presence of hydrogel showed progressive increases as compared to the corresponding unstressed and stressed plants cultivated without hydrogel addition. At 50% IWR, the most pronounced increases in all tested growth parameters were obtained by using 400 mg/L thiourea 16.62, 22.92, 21.88 and 23.03%, respectively in the presence of hydrogel as compared with the corresponding drought level control (50% IWR).

### Photosynthetic pigments

**Fig. 2:**
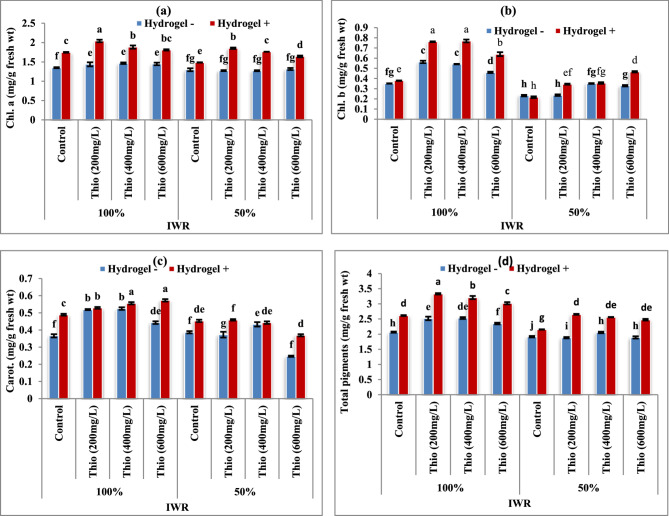
**a-d** Impacts of thiourea (Thio) on photosynthetic pigment derivatives of faba bean plants with various levels of irrigation water requirement (IWR) in the absence (-) and presence (+) of hydrogel. Data are means ± standard error of three replicates (*n* = 3). a, b, c,…. Columns with different letters are significantly differed at (*P* < 0.05)

According to the findings given (Fig. 2a-d), drought stress caused a significant (*P* < 0.05) decrease in total pigments (d), carotenoids (c), chlorophyll a (a), and chlorophyll b (b) in *Vicia faba* leaves. Meanwhile, the foliar spraying with thiourea significantly (*P* < 0.05) improved these photosynthetic pigments in contrast with the corresponding untreated plants. The addition of hydrogel to the experimental soil induced progressive increases in all photosynthetic pigments compared to stressed and unstressed plants. The maximum increases in total pigments were obtained in faba bean leaves by using the lowest concentration of thiourea (200 mg/L) and the percentages of increase were 21.50 and 21.74% at 100% IWR and 50% IWR, respectively in the presence of hydrogel in contrast with the corresponding untreated plants.

### Indole acetic acid (IAA) and phenol contents

**Fig. 3:**
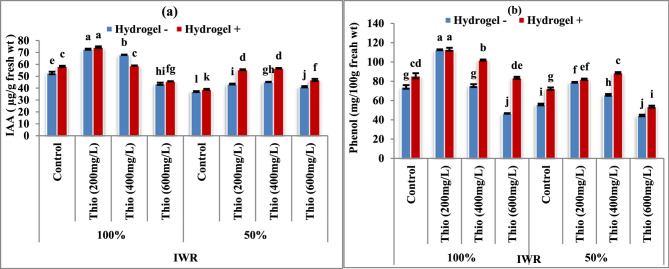
**a **& **b** Impact of thiourea (Thio) on (**a**) IAA and (**b**) phenol contents of faba bean plants with various levels of irrigation water requirement (IWR) in absence (-) and presence (+) of hydrogel. Data are means ± standard error of three replicates (*n* = 3).a, b, c,…. Columns with different letters are significantly differed at (*P* < 0.05)

It is apparent in Fig. 3a & b that in stressed plants (50% IWR), IAA (a) and phenol (b) content decreased significantly (*P* < 0.05) in the absence (-) and presence (+) of hydrogel in contrast with the corresponding untreated plants. Meantime, thiourea foliar spraying (200, 400 and 600 mg/L) increased significantly (*P* < 0.05) the IAA (Fig. 3a) and phenol (Fig. 3b) content in stressed and unstressed plants. The most pronounced increase was observed in response to the lowest concentration of thiourea at the rate of 200 mg/L in the presence and absence of hydrogel.

### Some osmoprotectant elements

**Fig. 4:**
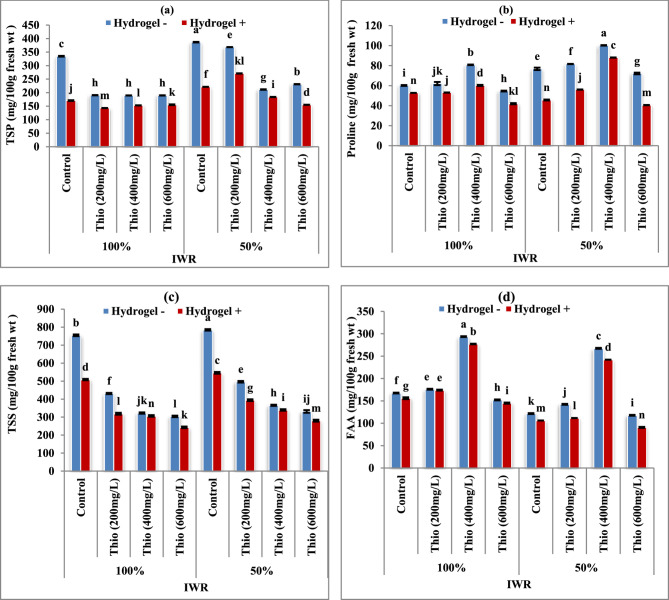
**a-d** Impact of thiourea (Thio) on osmoprotectant contents of faba bean plants with various levels of irrigation water requirement (IWR) in absence (-) and presence (+) of hydrogel. Data are means ± standard error of three replicates (*n* = 3). a, b, c,…. Columns with different letters are significantly differed at (*P* < 0.05)

Data in Fig. 4a-d showed the content of the osmoprotectant substance in the forms of (a) total soluble protein (TSP), (b) proline, (c) total soluble sugar (TSS), and (d) free amino acids (FAA) in absence (-) and presence (+) of hydrogel. In drought-stressed plants, it showed a significant increase (*P* < 0.05) in TSS, proline and TSP contents with and without hydrogel addition. Meanwhile, the other chemical constituents (FAA) were decreased significantly (*P* < 0.05). Thiourea foliar spraying at all rates reversed this result where TSS and TSP gradually decreased. Meanwhile, FAA and proline showed significant increases (*P* < 0.05) in drought-stressed or unstressed plants. The most noticeable rise in FAA and proline is shown in response to 400 mg/L thiourea.

### Some antioxidant enzymes

**Fig. 5:**
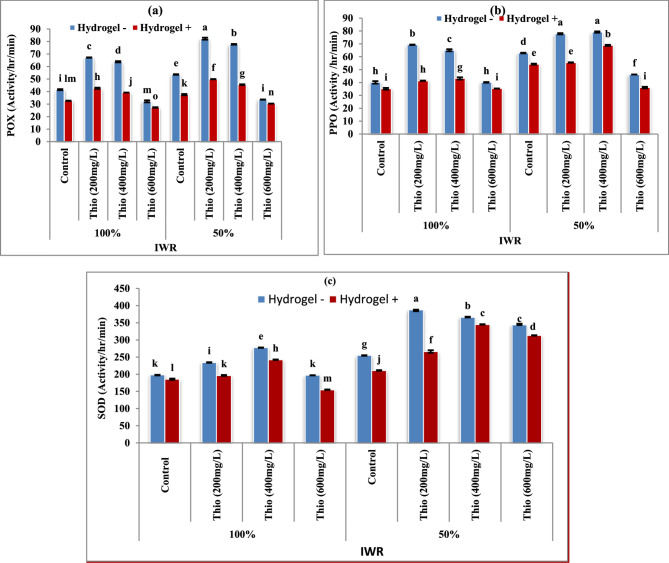
**a-c** Impacts of thiourea (Thio) on antioxidant enzymes of faba bean plants with various levels of irrigation water requirement (IWR) in absence (-) and presence (+) of hydrogel. Data are means ± standard error of three replicates (*n* = 3). a, b, c,…. Columns with different letters are significantly differed at (*P* < 0.05)

The current study’s findings (Fig. 5a-c) demonstrated that the antioxidant enzymes’ activity (a: peroxidase, POX; b: polyphenol oxidase, PPO and c: superoxide dismutase, SOD) in the shoots of faba bean plants exhibited to drought stress were significantly (*P* < 0.05) increased, in contrast with those of the untreated plants. Also, there are significant (*P* < 0.05) increases in all tested antioxidant enzyme content (POX, PPO and SOD) with increasing thiourea concentration (200 and 400 mg/L) in faba bean plants in the absence and presence of hydrogel in both drought-stressed and unstressed plants.

### Yield and yield components

Table 3 results revealed that plants exposed to 50% IWR saw a significant (*P* < 0.05) decline in all yield traits (shoot length, plant weight, number of pods/plant, weight of pods/plant, weight of seeds/p, number of seeds/plant, weight of pods/area, weight of plant/area and weight of seeds/area) in contrast with the untreated plants. A positive effect for various concentrations of thiourea was detected in the tested yield parameters of faba bean plants which significantly (*P* < 0.05) increased in contrast with the corresponding untreated plant levels. It is worth mentioning that the addition of hydrogel to the experimental soil enhanced these increments significantly (*P* < 0.05) in stressed and unstressed plants. In stressed plants (50%) with amended hydrogel, the percentage of increment in number of pods/plant, weight of pods/plant, number of seeds/plant, weight of seeds/plant, seed yield (ton/ha), biological yield and straw yield were 31.25, 28.83, 10.51, 23.73, 58.25, 58.25, 59.29 and 60.23% compared to the plants cultivated without hydrogel. From the data in the same tables, it is obvious that the most pronounced increase in the tested yield parameters was observed at the lowest concentration of thiourea (200 mg/L) under unstressed conditions (100% IWR) and the middle concentration (400 mg/L) under low water used (50%) both with hydrogel addition to the soil.

**Table 3:** Impact of thiourea and /or hydrogel with water regime on yield traits of bean plants

IWR (%)	Thiourea (mg/L)	Shoot length		Plant weight		No. of pods/plant		Wt. of pods/plant	
		Hydrogel -	Hydrogel +	Hydrogel -	Hydrogel +	Hydrogel -	Hydrogel +	Hydrogel -	Hydrogel +
**100**	**Control**	112.3 ± 0.33^de^	115.3 ± 0.88^cd^	110.2 ± 0.84^h^	119.9 ± 0.30^f^	24.33 ± 0.88^fg^	25.00 ± 1.15^f^	69.13 ± 0.83^f^	93.6 ± 0.465^c^
	**200**	118.0 ± 0.58^e^	122.3 ± 1.20^b^	123.9 ± 0.07^e^	135.9 ± 0.79^b^	31.67 ± 0.67^bc^	37.00 ± 0.58^a^	91.85 ± 0.49^d^	123.72 ± 0.41^a^
	**400**	119.0 ± 0.58^b^	131.0 ± 1.00^a^	135.9 ± 0.83^b^	166.6 ± 0.62^a^	34.00 ± 1.00^b^	32.31 ± 0.88^bc^	68.71 ± 0.41^fg^	97.79 ± 0.89^b^
	**600**	103.7 ± 0.67^g^	112.7 ± 0.33^de^	114.5 ± 0.29^g^	134.2 ± 1.42^b^	21.00 ± 0.00^h^	26.67 ± 1.86^ef^	67.14 ± 0.57^g^	69.53 ± 0.33^f^
**50**	**Control**	74.0 ± 1.00^l^	81.0 ± 1.15^k^	61.9 ± 1.18^m^	88.4 ± 0.46^l^	16.00 ± 0.58^i^	21.00 ± 0.58^h^	45.48 ± 0.62^l^	58.59 ± 0.42^j^
	**200**	89.0 ± 0.58^j^	94.7 ± 0.33^i^	88.1 ± 0.44^l^	126.0 ± 0.53^d^	22.33 ± 0.33^gh^	27.67 ± 0.88^de^	63.07 ± 0.35^h^	74.20 ± 0.60^e^
	**400**	90.7 ± 2.40^j^	108.7 ± 0.67^f^	104.9 ± 0.48^j^	131.6 ± 0.51^c^	25.67 ± 0.67^ef^	30.00 ± 0.58^cd^	68.36 ± 0.34^fg^	91.37 ± 0.69^d^
	**600**	82.3 ± 1.45^k^	100.0 ± 0.58^h^	103.1 ± 0.80^jk^	101.9 ± 0.57^k^	17.00 ± 0.67^i^	20.33 ± 0.58^h^	51.04 ± 0.41^k^	61.25 ± 0.77^i^

**IWR **	**Thiourea (mg/L)**	**No. of seeds /plant**		**Wt. of seeds /plant**		**Seed yield (Kg/ha)**		**Biological yield (Kg/ha)**		**Straw yield (Kg/ha)**	
**(%)**		**Hydrogel -**	**Hydrogel +**	**Hydrogel -**	**Hydrogel +**	**Hydrogel -**	**Hydrogel +**	**Hydrogel -**	**Hydrogel +**	**Hydrogel -**	**Hydrogel +**
**100**	**Control**	73.00±0.83^e^	80.67±0.46^d^	60.67±0.84^d^	75.07±0.56^b^	1330±2.78^l^	2110±2.08^d^	2710±1.86^l^	4320±1.94^c^	1380±3.56^m^	2290±0.97^c^
	**200**	84.00±0.49^c^	93.33±0.41^a^	68.73±0.72^c^	97.12±0.81^a^	1650±1.66^i^	2490±1.94^a^	3230±1.88^i^	5070±2.73^a^	1580±0.99^i^	2580±4.54^a^
	**400**	90.00±0.41^b^	91.33±0.89^ab^	51.60±0.64^f^	75.55±0.35^b^	1670±1.04^h^	2450±1.80^b^	3400±1.95^f^	4690±1.88^b^	1730±2.78^f^	2230±0.84^b^
	**600**	89.33±0.57^b^	90.33±0.33^ab^	41.85±0.71^k^	44.81±0.33^ij^	1850±2.15^e^	2220±1.52^c^	3750±0.99^d^	4320±1.72^c^	1900±3.65^e^	2090±0.94^d^
**50**	**Control**	34.67±0.62^i^	42.67±0.42^h^	35.20±0.87^l^	45.80±0.62^hi^	860±1.69^o^	1420±1.45^j^	2020±0.87^o^	3000±0.98^j^	1160±0.87^n^	1580±1.86^i^
	**200**	68.00±0.35^f^	66.00±0.60^f^	47.27±0.54^h^	58.18±0.27^e^	1100±1.45^n^	1660±2.15^h^	2620±1.97^n^	3340±1.99^g^	1510±0.87^k^	1680±0.53^h^
	**400**	54.00±0.34^g^	72.67±0.69^e^	49.79±0.27^g^	61.50±0.81^d^	1300±1.94^m^	1810±1.87^f^	2680±1.75^m^	3520±1.86^e^	1380±0.97^m^	1710±0.96^g^
	**600**	51.33±0.41^g^	51.67±0.77^g^	36.03±0.36^l^	43.83±0.37^j^	1410±1.84^k^	1780±1.73^g^	2830±1.54^k^	3310±1.66^h^	1420±0.75^k^	1530±0.71^j^

Data are means ± standard error of three replicates (*n* = 3)

a, b, c,…. Columns with different letters are significantly differed at (*P*<0.05)

(-) means absent of hydrogel, (+) means present of hydrogel

### Nutritive value of seeds

The records presented in Table 4 revealed the impact of foliar application of faba bean with thiourea in the absence and presence of hydrogel at two levels of irrigation water requirements (100 & 50% IWR) on seed quality (Nitrogen, phosphorous, potassium, protein, carbohydrate percentages, and protein yield). It was found that water stress at 50% IWR induced significant (*P* < 0.05) decreases of all previous traits in the yielded seeds compared with controls (100% IWR). Meanwhile, thiourea foliar spraying at all rates under the two levels of IWR induced an increase in all the macronutrients mentioned above, carbohydrate, and protein content in comparison with the corresponding control. The amendment of hydrogel improved seed quality compared with its absence. It is obvious from the table that the % of increases in protein yield and carbohydrate content under drought stress (50% IWR) and in the presence of hydrogel were 26.88, 29.84 & 14.75 and in response to 200, 400, and 600 mg/L thiourea were 6.66, 10.00 & 2.32, respectively. It is clear that 400 mg/L thiourea was the most effective treatment.

**Table 4:** Impact of thiourea and/or hydrogel with water regime on seed quality of bean plants

IWR (%)	Thiourea (mg/L)	N %		P %		K %		Protein %		Protein yield (Kg/ha)		Carb. %	
		Hydrogel -	Hydrogel +	Hydrogel -	Hydrogel +	Hydrogel -	Hydrogel +	Hydrogel -	Hydrogel +	Hydrogel -	Hydrogel +	Hydrogel -	Hydrogel +
**100**	**Control**	3.598 ± 0.01^h^	3.931 ± 0.02^d^	0.06 ± 0.001^ef^	0.069 ± 0.002^cd^	0.99 ± 0.003^d^	1.01 ± 0.005^c^	22.49 ± 0.08^h^	24.57 ± 0.13^d^	300.02 ± 0.98^i^	518.74 ± 2.53^c^	53.65 ± 0.98^fg^	60.39 ± 0.79^cd^
	**200**	3.721 ± 0.01^f^	4.284 ± 0.01^a^	0.066 ± 0.002^de^	0.115 ± 0.003^a^	0.99 ± 0.005^d^	1.1 ± 0.003^b^	23.27 ± 0.05^f^	26.78 ± 0.06^a^	389.98 ± 1.08^f^	666.35 ± 1.84^a^	60.53 ± 0.85^cd^	66.48 ± 0.69^b^
	**400**	4.024 ± 0.02^c^	4.181 ± 0.03^b^	0.068 ± 0.002^cd^	0.085 ± 0.003^b^	1.02 ± 0.005^c^	1.16 ± 0.002^a^	25.15 ± 0.13^c^	26.13 ± 0.20^b^	418.74 ± 1.99^d^	641.22 ± 5.21^b^	61.71 ± 0.99^c^	69.98 ± 0.95^a^
	**600**	3.404 ± 0.01^i^	3.773 ± 0.01^e^	0.054 ± 0,001^fg^	0.069 ± 0.001^cd^	0.96 ± 0.002^e^	1.1 ± 0.002^b^	21.28 ± 0.04^i^	23.6 ± 0.07^e^	393.69 ± 0.71^f^	517.60 ± 1.38^c^	59.79 ± 0.87^d^	69.37 ± 0.85^a^
**50**	**Control**	3.453 ± 0.02^i^	3.582 ± 0.04^h^	0.046 ± 0.002^h^	0.088 ± 0.002^g^	0.88 ± 0.004^g^	0.89 ± 0.005^g^	21.58 ± 0.10^i^	22.39 ± 0.22^h^	184.55 ± 1.09^l^	318.89 ± 3.31^h^	48.56 ± 0.88^k^	50.48 ± 0.86^j^
	**200**	3.592 ± 0.01^h^	3.899 ± 0.02^d^	0.053 ± 0.002^g^	0.073 ± 0.001^c^	0.26 ± 0.004^j^	0.93 ± 0.005^f^	22.45 ± 0.05^h^	24.37 ± 0.14^d^	247.60 ± 0.78^k^	404.60 ± 2.13^e^	51.98 ± 1.04^hi^	53.84 ± 1.04^f^
	**400**	3.555 ± 0.04^h^	3.658 ± 0.01^g^	0.06 ± 0.001^ef^	0.066 ± 0.001^de^	0.353 ± 0.003^j^	0.85 ± 0.004^h^	22.22 ± 0.22^h^	22.86 ± 0.04^g^	289.480 ± 3.15^j^	414.05 ± 0.69^d^	52.30 ± 0.57^gh^	55.53 ± 0.87^e^
	**600**	3.288 ± 0.03^j^	3.295 ± 0.04^j^	0.017 ± 0.004^i^	0.04 ± 0.003^h^	0.25 ± 0.001^j^	0.74 ± 0.001^i^	20.55 ± 0.12^j^	20.59 ± 0.16^j^	289.39 ± 1.94^j^	365.92 ± 2.60^g^	50.79 ± 0.77^ij^	51.65 ± 0.99^hij^

Data are means ± standard error of three replicates (*n* = 3)

a, b, c,…. Columns with different letters are significantly differed at (*P*<0.05)

(-) means absent of hydrogel, (+) means present of hydrogel

### Water productivity (WP)

**Fig. 6:**
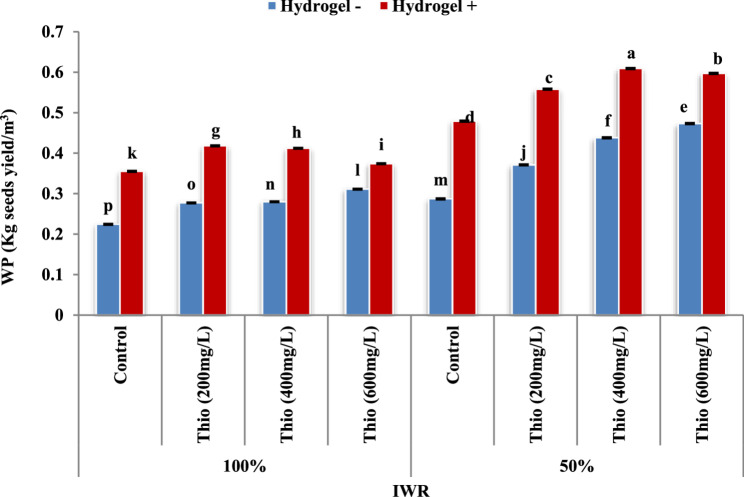
Impact of thiourea without (-) and with (+) hydrogel on water productivity (WP; Kg seed yield/m^3^) of faba bean plants at two levels of irrigation water requirement (IWR). Data are means ± standard error of three replicates (*n* = 3). a, b, c,…. Columns with different letters are significantly differed at (*P* < 0.05)

The findings in Figure (6) showed that the treatment of faba bean plants with thiourea (200, 400 and 600 mg/L) significantly (*P* ≤ 0.05) increased WP (The ratio of crop yield divided by water consumed; Kg seed yield/m^3^). The WP increased from 0.355 (control) to 0.418, 0.412 and 0.374 Kg seed yield/m^3^ (at 100% IWR) with the % of increase 17.7, 16.1 and 5.4, respectively. Meanwhile it increased from 0.479 (control) to 0.558, 0.609, and 0.597 Kg seed yield/m^3^ (at 50% IWR) with the % of increase 16.5, 27.1 and 24.6, respectively in the presence of hydrogel. The positive enhancement in WP with thiourea application was achieved when plants exposed to drought stress (50% of IWR) more than those received 100% of IWR. The most pronounced rise in WP was seen in the presence of hydrogel with thiourea (400 mg/L).

### Principal component analysis (PCA) and pearson correlations

**Fig. 7:**
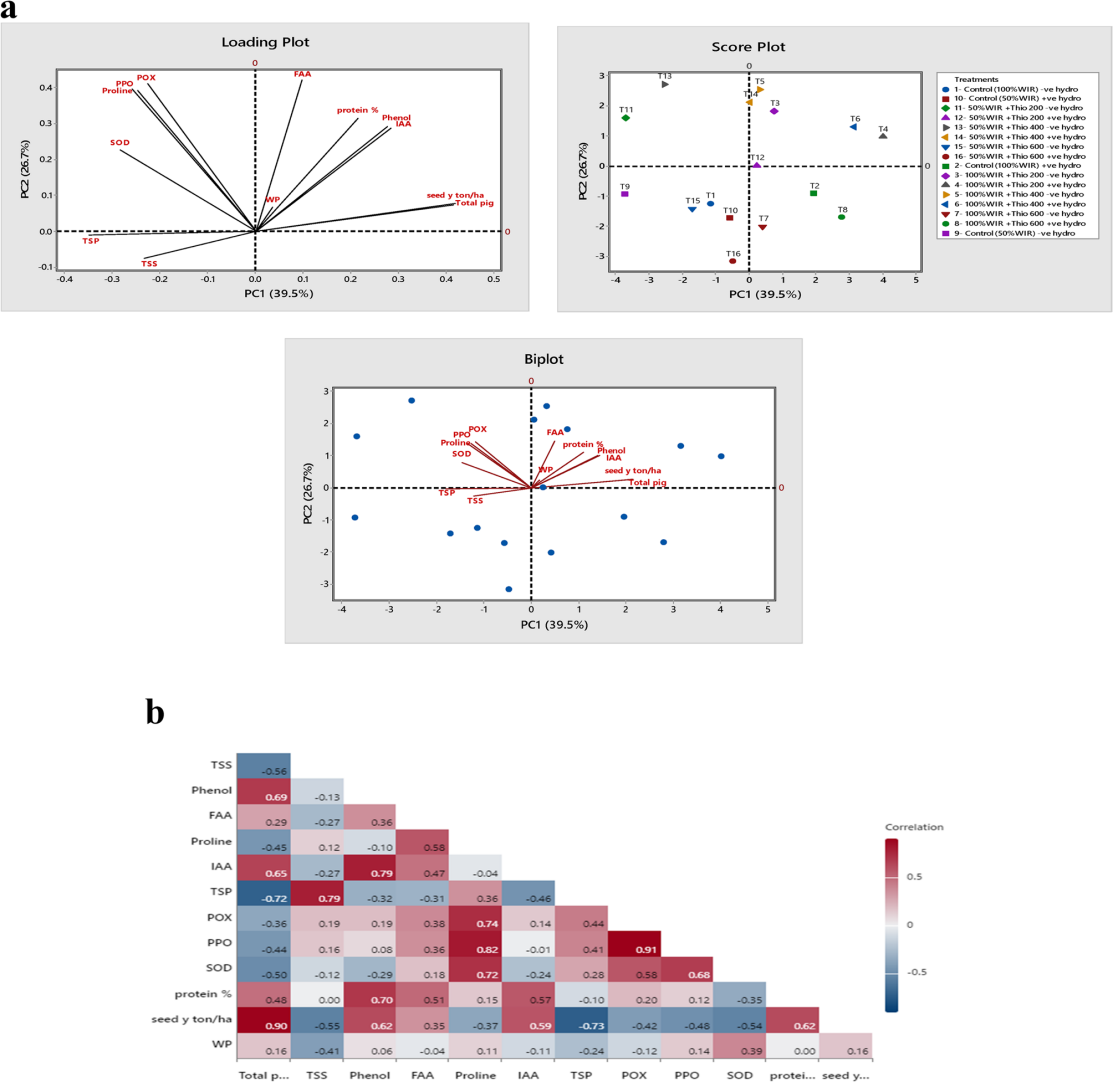
**a** Principal component analysis (PCA) for some biochemical, and yield traits of faba bean plants with thiourea treatments in the absence (-) and presence (+) of hydrogel under various levels of irrigation water requirement (IWR). **b** Heat map of Pearson correlation analysis for some biochemical, and yield traits of faba bean plants with thiourea treatments in the absence (-) and presence (+) of hydrogel under various levels of water irrigation requirement (IWR). Blue and red colors represent negative and positive correlations, respectively

This technique is used in data analysis in the form of principal components (PCs), which represent the linear combinations of the original variables, where the variance of the data is maximized. This test carried out for finding out the relationship between seed yield (as the crop economic return element) and the studied physiological parameters. In this study, the first PC (PC1) explains the highest (39.5%) total variance, and the second PC (PC2) explains the lowest significant amount of variance (26.7%). Both PC1 and PC2 together explain more than 65.12% of the total variance (Fig. 7a). The antioxidant enzymes (POX, PPO, and SOD) and proline were associated with 50% IWR when 200 and 400 mg/L thiourea was applied without hydrogel addition. The contents of FAA, protein %, phenol, IAA, total photosynthetic pigments, water productivity and seed yield (ton/ha) were associated with 100% IWR with 200 and 400 mg/L thiourea in absence and presence of hydrogel amendment. It was also associated with 50% IWR + 200 and 400 mg/L thiourea with hydrogel addition. Meanwhile, the osmoprotectants (TSS and TSP) has a high correlation with control (50 and 100% IWR with and without hydrogel) and also, the treatment of 50% IWR with 600 mg/L thiourea without hydrogel amendment.

Numerous significant connections between the physiological measures and the yield of faba beans were shown by heat map correlation analysis (Fig. 7b). From the data, there were strong positive correlations observed between seed yield (Kg/ha) and all of the tested physiological parameters; total pigments (*r* = 0.90), phenols (*r* = 0.62), IAA (*r* = 0.59) and protein (*r* = 0.62) and also between the content of antioxidant enzymes (POX), PPO and SOD with proline (*r* = 0.74), (*r* = 0.82) and (*r* = 0.72), respectively. Meanwhile, a negative relationship was observed between seed yield (Kg/ha) and TSS (*r* = −0.55), proline (*r* = −0.37) and TSP (*r* = −0.73), POX (−0.42), PPO (*r* = −0.48) and SOD (*r* = −0.54).

Concerning protein content in the yielded seeds (as an indicator of seed quality), a positive correlated with total pigments (*r* = 0.48), phenols (*r* = 0.70), FAA (*r* = 0.51) and IAA (*r* = 0.57), while it had weak positive correlation with proline (*r* = 0.15), POX (*r* = 0.20) and PPO (*r* = 0.12). Meanwhile, a negative correlation was observed with TSS, TSP, and SOD (*r* = −0.01, −0.10 and − 0.35, respectively).

## Discussion

The tested growth criteria of faba bean plants (Table 2) decreased (*P* < 0.05) in response to drought stress. As evident from the literature, irrigation water deficit induced severe effects on the plant morphological, physiological, biochemical, and molecular attributes. It induced a disruption in photosynthetic activity via altering plant water relationships, photosynthetic pigments, mineral requirements, oxidative damage to macromolecules, membrane degradation, and decreases in enzyme activities [39]. Such effect may be attributed to the effect of drought which decreases gas exchange, water relation, and rooting attributes, and shoots dry mass of sesame plants, while it increases substomatal CO_2_ concentration, proline concentration, and root/shoot dry mass [40]. They attributed the increase in root/shoot dry mass to the cumulative root length, root length density, specific root length, and root surface upon experiencing stress.

On contrast, thiourea and hydrogel application mitigate the harmful effect of drought stress which stimulate the growth of faba bean plants. This result may be due to the increase in photosynthetic pigments (Fig. 2) and growth promotor; IAA (Fig. 3). Thiourea increased cell division and targeted the meristematic activity of apical tissues, which led to longer shoots and more cells and consequently increase the fresh and dry weights of shoots [41]. These effects can be explained by the opinion of Sahu [42] who stated that thiourea is regulating and improving phloem water transport in plants, thus preserve cellular water homeostasis and enhance plant acclimation to drought stress. Albalasmeh et al. [43] found that hydrogel at 0.27% and 0.33% increased the soil available water for corn plants by 35 and 49%, respectively and also water use efficiency was increased by 28 and 32% in sandy and silty clay loam soil, respectively.

The suppression of photosynthetic activity (Fig. 2) as a result of drought stress may involve different mechanisms, such as stomatal closure [44], chlorophyll degradation and Chl *b* is converted into Chl *a*, leading to an increase in Chl *a*/*b* ratio with stress conditions [45]. As reviewed and discussed by Askari and Ehsanzadeh [45] under such circumstances (ratio of carotenoids/cholorophyll increases), as the reduction in chlorophyll content leads to reduction in absorbed photons and considering the antioxidative function of carotenoids, enhancement of the photoprotective and antioxidative capacity of the leaves possibly take place. Thiourea and hydrogel were used to mitigate the lowering effect on photosynthetic activity, which was enhanced in treated plants under drought or non-stressful conditions. The study’s findings may be interpreted by the effective function of thiourea in reducing oxidative damage to the photosynthetic machinery and increases the amount of chlorophylls [46]. The enhancement of photosynthetic pigments due to thiourea treatments may be due to the promotion in high efficiency of PSI and PSII [47]. Also, Sharma et al. [48] added that the action of thiourea treatments may have targeted the meristematic activity of apical tissues, exhibiting stimulatory effects on cell division that lead to an increase in shoot length and cell quantity, ultimately improving the fresh and dry weight of the shoots and roots. Concerning the application of water-retaining polymer (hydrogel), Abd El-Aziz et al. [49] and Soliman et al. [50] found that it increased the content of chl a, chl b, carotenoids in leaves of *Calendula officinalis* plants. They added that such effect due to hydrogel polymer’s performance may be ascribed to the reduction in chlorophyll degradation or enhanced its biosynthesis because of adequate water (Increased soil moisture) and nutrients supply to the plants.

Phenolics are known to be either directly or indirectly involved in plant mechanism, through oxidation of phenols and activation of plant defense enzymes, in turn, to scavenge the oxidative damage substances, e.g. H_2_O_2_. Such compound triggers several physiological and molecular processes that pointer the production of numerous defense elements and enzymes [45]. The thiourea and hydrogel addition’s stimulating total phenolic and IAA levels of faba bean plants (Fig. 3). These observation are consistent with Baqer et al. [51] who found that foliar application of thiourea amended the antioxidant compound synthesis of wheat plants (Phenols, riboflavin, anthocyanins, and ascorbic acid). Besharati et al. [52] showed increased total phenolic content and antioxidant activity by hydrogel treatment in *Hibiscus sabdariffa* plants.

Under drought stress, most tested osmoprotectants (TSS, proline and TSP) as showed in Fig. 4 enhanced in faba bean leaves to increase the osmosis consequently the plant’s ability to absorb water. The osmoprotectants were necessary function for carbon storage, osmotic correction, and increasing radical scavenging under water stress conditions [53]. Increasing these elements enhanced cells’ capacity to respond to various stressors by increasing the cytoplasmic osmotic pressure and stabilizing proteins and membranes that maintain the comparatively greater water content required for plant growth and cell function [54]. The enhancement of proline, which declined due to drought stress, could enhance the osmotic pressure of cytoplasm and boost the water potential of plant tissues for recovery from stress [45]. Concerning the effect of tested materials, thiourea significantly enhanced the FAA contents of wheat plants [55, 56] and hydrogel treatments (0.4 and 0.6%) increased the contents of phenols and FAA in *Calendula officinalis* plants [50]. Additionally, it has been proven that proline protects the cell machinery from malfunction induced by lipid peroxidation and detoxifies the membrane from damaging ROS [57]. They concluded that proline plays a variety of roles throughout increases the rate of photosynthesis which interacts with many different signaling molecules, including plant growth regulators (IAA), activates stress signaling (TSS, TSP, FAA and proline) and aids in the up-regulation of stress-related genes. These effects protects the photosynthetic apparatus, helps maintain the cellular redox balance [58, 59].

The data of antioxidant enzymes’ activity (POX, PPO and SOD) in the shoots of faba bean plants in response to thiourea and hydrogel application presented in Fig. 5 showed and improvement which can be supported by the assumption of protecting plants against oxidative damage [60]. Our results were confirmed by Fiaz et al. [61] who found that under drought stress (60% Field Capacity; FC) with thiourea foliar application at 1000 mg/L enhanced the contents of antioxidant enzymes catalase, peroxidase, and superoxide dismutase by 40, 13, and 30%, respectively in Roshini and Chandni flax cultivars. Thiourea can completely prevent H_2_O_2_-promoted senescence and the H_2_O_2_-caused reduction in activities of SOD and peroxidase (APX) in light and darkness [62]. Concerning the effect of hydrogel addition, Soliman et al. [50] found that hydrogel alone at 0.6% (w/w) increased the antioxidant enzymes activity (Catalase, Superoxide dismutase, and Peroxidase) with or without stress.

Decreasing irrigation water requirement (50% IWR) significantly (*P* < 0.05) decreased yield traits of faba bean plants (Table 3). Meanwhile, thiourea and hydrogel at different concentrations increased all yield and its components in stressed and unstressed plants. The significant increase in yield and yield attributes of faba bean plants due to treatment with thiourea and soil hydrogel addition may be due to the increase in morphological criteria and plant water content (Table 2), photosynthetic pigments (Fig. 2), the content of IAA and phenols (Fig. 3), proline content (Fig. 4), and the antioxidant enzymes (Fig. 5). This trend supported by the results of Ahmad et al. [62] on Camelina and [63] on canola plants. Hydrogel at rate of 6 g/m^2^ had superiority in the number of leaves per plant, number of bolls per plant, and seed cotton weight per plant [11]. Also, in a trial conducted by Waly et al. [15] with two crops (Rice in the summer and barley in the winter), it was found that adding 1% hydrogel to the soil weight of the rice crop produced taller plants with the largest leaf area, more tillers per pot, more grains per panicle, heavier panicles, higher grain and biological yield per pot and a higher harvest index, than other treatments. Meanwhile, barley plants treated with 0.2% hydrogel resulted in the greatest number of spikes/pot, the heaviest grain weight, the highest grain yields, the highest harvest index, and the highest biological yields. They added that the increase in yield due to hydrogel addition may be due to increasing the soil water retention capacity and plant water potential.

The data obtained herein (Table 4) revealed a significant increase in seed quality (Nitrogen, phosphorous, potassium, protein, carbohydrate percentages, and protein yield) in response to thiourea and hydrogel treatments in both level of IWR. The role of thiourea in increasing total protein, carbohydrates and total fats that stimulating productivity may be attributed to the increased rhizosphere nutrient absorption and enhanced plant metabolic activities [64]. This action may be connected to the metabolic role of the SH-group in the biochemistry and physiology of roots that promoted the growth and development of plants. Also, Choudhary et al. [65] found that thiourea foliar spray increased N, P and K content and consequently protein content in the yielded seeds. In this concern, Srivastava et al. [66] explained that thiourea regulated the manufacture of hormones, particularly auxins of *Brassica juncea* under salinity stress. They also mentioned that thiourea enhanced microbial population in the soil, facilitates the release of essential nutrients and water homeostasis of the root during salinity stress. Concerning the effect of hydrogel, the oil yield as well as nitrogen, phosphorus, and potassium fertilizers use efficiencies were boosted in flax plants at 75% IWR when hydrogel used with farmyard manure application [67]. Also, Waly et al. [15] discovered that the protein % in yielded grains increased with the amendment of 1% hydrogel to the soil (weight/weight) of rice in the summer and barley in the winter.

Increasing water productivity considered paramount to meet future humanity food demand especially with water scarcity resulted from weather changes. There are interrelated linkages between crop water productivity and applied water productivity as affected by irrigation management decisions to make economic decision under water limiting conditions [68]. Water productivity (WP) of faba bean plants (Fig. 6) increased in response to thiourea treatments in the presence and absence of hydrogel at both levels of IWR. Hydrogel addition empowered WP in the control and thiourea treated plants and its effect was more noticeable when plants received only 50% of its IWR. Thiourea boosted net photosynthetic rate, nitrate reductase activity and soluble protein in the plants, thus enhancing water productivity of rain-fed cluster bean [69]. Concerning hydrogel application, a conservation of about 14.8% from applied water was detected with an improvement in rice grain yield by about 16.5% and water productivity increased from 0.32 to 0.48 kg/m^3^ compared to for those without hydrogel treatment [70]. This improvement in water productivity may be due to the role of hydrogel polymers in improving soil moisture content that keeps its wetting for longer period due to moisture retention. This action also raised soil water swelling and releasing capacity against its pressure [12]. So far, reaching the best WP may lead to suboptimal economic outcomes and supply enough food resources.

The results showed in figure (7b) indicated that yielded seeds quality in terms of protein content had a positive correlation relationship with total pigments, phenols content, FAA and IAA which can be considered the most significant contributory traits for seed yield/plant. These results are supported by the findings of El-Bassiouny et al. [71] on flax. These results suggested that selection of such treatments under similar environmental circumstances would be quite effective for improving seed yield in faba bean.

## Conclusion

As evident from the obtained results herein, there was an improvement in water absorption by 45.53%. This action may be considered the reason behind the improvement in weight of seeds/plant, seed yield, biological yield and straw yield by, 58.25, 58.25, 59.29 and 60.23%, respectively. These results extended herein achieved when thiourea (400 mg/L) and hydrogel (75 kg/ha) were applied. So, it is worthy to mention that, under drought conditions (50% IWR), foliar spray with thiourea and hydrogel amendment to sandy soil not only positively affected water potential, osmolytes (TSS, TSP, proline, FAA, IAA and phenol), chlorophyll, and carotenoids contents, but also significantly changed the activities of antioxidant enzymes (POX, PPO and SOD) that all these traits enabled faba bean plants to withstand drought and increased productivity (Quantity and quality). The obtained results recommended the application of thiourea with the hydrogel addition to lessen the detrimental effects of shortage of irrigation water in newly reclaimed sandy soils.

## Data Availability

The corresponding author can provide the datasets used and/or analyzed during the current work upon proper request.
